# Comparative metabolic profiling of posterior parietal cortex, amygdala, and hippocampus in conditioned fear memory

**DOI:** 10.1186/s13041-021-00863-x

**Published:** 2021-10-06

**Authors:** Yoonjeong Jeon, Yun Lim, Jiwoo Yeom, Eun-Kyoung Kim

**Affiliations:** 1 Department of Brain and Cognitive Sciences, Daegu Gyeongbuk Institute of Science and Technology (DGIST), Daegu, 42988 Republic of Korea; 2 Neurometabolomics Research Center, Daegu Gyeongbuk Institute of Science and Technology (DGIST), Daegu, 42988 Republic of Korea

**Keywords:** Conditioned fear memory, Metabolomics, Posterior parietal cortex, Amygdala, Hippocampus

## Abstract

Fear conditioning and retrieval are suitable models to investigate the biological basis of various mental disorders. Hippocampus and amygdala neurons consolidate conditioned stimulus (CS)-dependent fear memory. Posterior parietal cortex is considered important for the CS-dependent conditioning and retrieval of fear memory. Metabolomic screening among functionally related brain areas provides molecular signatures and biomarkers to improve the treatment of psychopathologies. Herein, we analyzed and compared changes of metabolites in the hippocampus, amygdala, and posterior parietal cortex under the fear retrieval condition. Metabolite profiles of posterior parietal cortex and amygdala were similarly changed after fear memory retrieval. While the retrieval of fear memory perturbed various metabolic pathways, most metabolic pathways that overlapped among the three brain regions had high ranks in the enrichment analysis of posterior parietal cortex. In posterior parietal cortex, the most perturbed pathways were pantothenate and CoA biosynthesis, purine metabolism, glutathione metabolism, and NAD^+^ dependent signaling. Metabolites of posterior parietal cortex including 4′-phosphopantetheine, xanthine, glutathione, ADP-ribose, ADP-ribose 2′-phosphate, and cyclic ADP-ribose were significantly regulated in these metabolic pathways. These results point to the importance of metabolites of posterior parietal cortex in conditioned fear memory retrieval and may provide potential biomarker candidates for traumatic memory-related mental disorders.

**Supplementary Information:**

The online version contains supplementary material available at 10.1186/s13041-021-00863-x.

## Introduction

The increase in the prevalence of mental illnesses has prompted extensive studies aimed at understanding their etiology and pathophysiology, and improving treatment [1, 2]. People exposed to various traumatic situations are at a great risk for developing several psychiatric conditions including anxiety, depression, and post-traumatic stress disorder (PTSD) [3, 4]. Aversive experiences of stimuli found in a specific situation set the occasion for robust fear of environmental contexts or cues paired with these events in humans and other animals [5, 6]. Many aspects of traumatic memories such as fear memory can be studied using Pavlovian fear conditioning paradigms, in which animals learn to associate a conditioned stimulus (CS) including contexts (chamber, cage, etc.) and cues (light, tone, odor, etc.) with an unconditioned stimulus (US) such as a mild foot shock [7–9]. After training, the cue and the context in which an animal was exposed can produce a fear response such as freezing behavior [10].

To understand learning processes including fear conditioning, retrieval, extinction, and renewal, the neural mechanisms by which CS and US representations are encoded in the brain have been investigated. It is well established that the context is processed within the hippocampal–cortical networks, whereas the cue and US are processed within the basolateral amygdala (AMG), whose output contributes to conditioned fear [11–14]. In fear learning processes, direct projections from the hippocampus (HPC) to AMG and indirect projections between the HPC and AMG via the prefrontal cortex (PFC) mediate fear responses [15–17]. Especially, ventral hippocampal CA1 projections to the basal AMG are necessary for encoding and retrieval of contextual fear memory [18]. Medial PFC is gradually involved in remote fear memory retrieval [19]. Although the AMG, HPC, and PFC are known as key regions for the regulation of conditioned fear memory, other brain regions that mediate CS-dependent modulation of the fear response have not been fully investigated.

The posterior parietal cortex (PPC) receives various sensory and cognitive stimuli, integrates multisensory signals [20–23], processes sensory information, and transforms this information into behavioral activity [24–26]. Cognitive processes are affected by the response of PPC neurons [27, 28]. Additionally, PPC neurons are activated in contextual fear conditioning and reactivated by fear memory retrieval [29]. These studies support the idea that the PPC has roles in memory formation and processing.

Metabolomics, a qualitative and quantitative analysis of small molecules present in biological samples, has been increasingly used as a tool to discover and develop biomarkers [30–32]. A number of attempts have been made to identify biomarkers of traumatic memory-related mental disorders. For example, using metabolic profiling of prefrontal extracellular fluid, metabolites such as xanthurenic acid, glucose-1-phosphate, sarcosine, and spermidine have been found to predict long-term PTSD-like symptoms [33]. In a study of social defeats (used as a conditioned fear model), the levels of cystine which is converted to cysteine, and fumarate were increased in the nucleus accumbens of stress-resistant and dominant animals, respectively [34]. In the PFC, the level of methionine was also elevated in dominant animals [34]. These studies suggest these metabolites as therapeutic biomarkers of stress-related mental disorders. However, potential biomarkers in other mental diseases remain unidentified. Therefore, identification of several neurochemicals in various brain tissues in the conditioned fear response could provide novel biomarkers that could be used to improve the treatment of psychopathologies associated with mental disorders [34, 35].

Here, we performed an unbiased metabolomics screening in the PPC, AMG, and HPC and identified metabolites relevant to conditioned fear memory. Our data demonstrated that the trends of altered metabolites and metabolic pathways in PPC and AMG were more similar to each other than that of HPC. Moreover, we found that several PPC-specific metabolites significantly changed after the retrieval of fear memory. These results may provide potential biomarker candidates for the diagnosis, risk assessment, and prevention of fear memory-related mental disorders via further validation.

## Results

### Contextual and cued fear conditioning, and retrieval test for mice

**Fig. 1:**
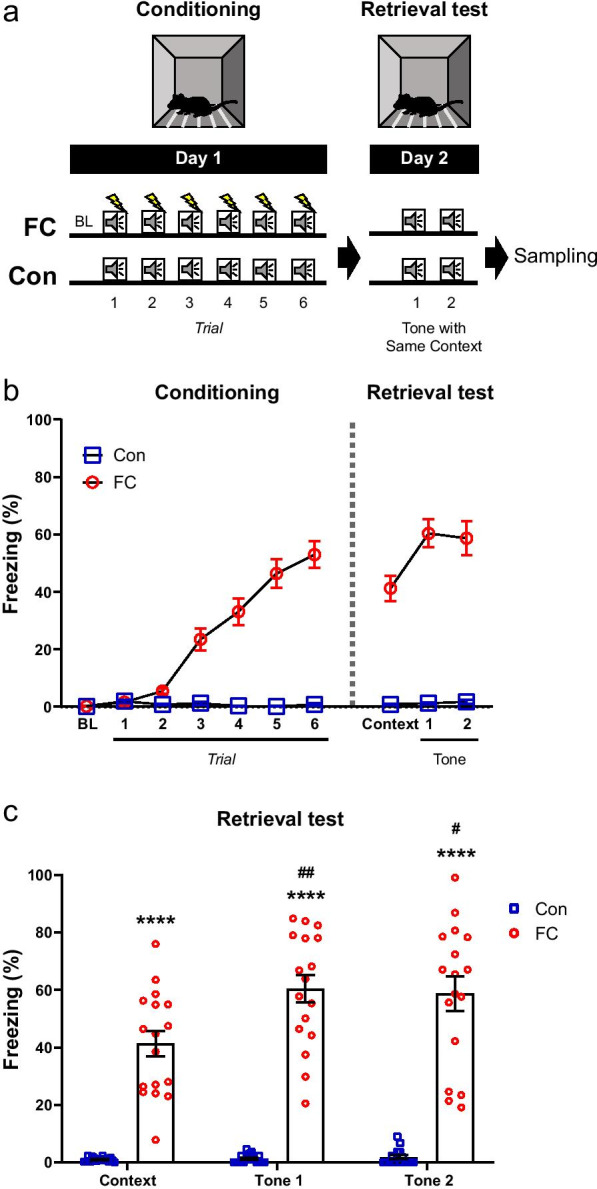
Behavior changes during fear conditioning and retrieval test. **a** Schematic diagram showing the experimental procedure for fear conditioning and retrieval test. **b** Percentage of freezing behavior across the conditioning and retrieval test sessions. Each dot represents the level of freezing when the tone stimulus was presented, except the first dot of each session, which shows the pre-tone baseline and the context test, respectively (Con, n = 16 mice; FC, n = 17 mice; two-way repeated-measures ANOVA, data are mean ± SEM). **c** Freezing in the context and tone stimulus during the retrieval test (Con-Context, n = 16 mice; FC-Context, n = 17 mice; Con-Tone 1, n = 16 mice; FC-Tone 1, n = 17 mice; Con-Tone 2, n = 16 mice; FC-Tone 2, n = 17 mice; one-way ANOVA, data are mean ± SEM; ****p < 0.0001 vs. Con, ^#^*p* < 0.05, ^##^*p* < 0.01 vs. Context). *BL* baseline, *Con* control, *Context* context test, *FC* fear conditioning

We first used a fear-conditioning paradigm to examine metabolic changes in brain regions induced by fear memory retrieval. Mice were subjected to a fear-conditioning task in which they learned to associate a cue (tone) with a US (foot shock) in a context. Mice in the fear-conditioning (FC) group were first exposed to six pairings of the tone and foot shock. Mice were then placed in the same context and received the tone twice without the foot shock during the next day. Control mice were exposed to the same task as the FC group but did not receive any foot shocks (Fig. 1a). During the task, conditioned fear responses were measured on the basis of the duration of freezing behavior (Fig. 1b, c).

Significant differences in freezing behavior were found between the control and FC groups during fear conditioning on day 1 [time × task interaction, F (6, 186) = 51.47, *p* < 0.0001; time, F (6, 186) = 49.30, *p* < 0.0001; task, F (1, 31) = 107.9, *p* < 0.0001, two-way repeated-measures analysis of variance (ANOVA) (Fig. 1b)]. On day 2, significant differences in fear responses to the tone and context were found between the control and FC groups during the retrieval test [time × task interaction, F (2, 62) = 8.67, *p* = 0.0005; time, F (2, 62) = 9.79, *p* = 0.0002; task, F (1, 31) = 135.6, *p* < 0.0001, two-way repeated-measures ANOVA (Fig. 1b)].

In the retrieval test, the levels of context-dependent freezing behavior were measured for the first 300 s (context test). To determine further changes in freezing by tone in the retrieval test, mice were then exposed to two tones in the same context. The level of freezing behavior was significantly higher in the FC group than the control group in the context only, and context and tone stimuli. After exposure to the tone with the same context, the levels of freezing behavior increased significantly in the FC group in comparison with the context test only (Fig. 1c).

### Metabolic profiling of three fear memory-related brain regions

**Fig. 2:**
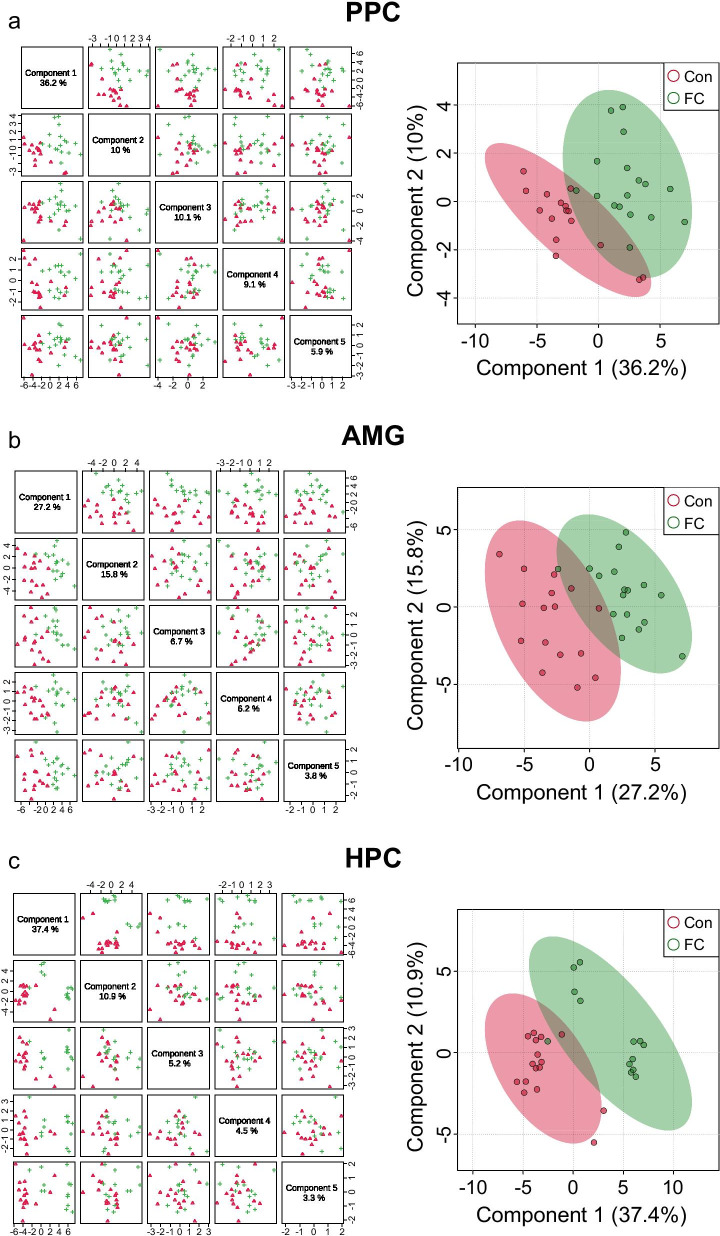
Pairwise score plots of selected components and 2D score plots in PLS-DA. **a** PPC. **b** AMG. **c** HPC. Each point corresponds to one mouse; shaded ellipses represent 95% confidence intervals (Con-PPC, n = 16 mice; FC-PPC, n = 17 mice; Con-AMG, n = 16 mice; FC-AMG, n = 17 mice; Con-HPC, n = 16 mice; FC-HPC, n = 13 mice). *Con* control, *FC* fear conditioning

To identify metabolites specific to conditioned fear memory in each brain region, we performed untargeted metabolite profiling of PPC, AMG, and HPC samples and focused on differences between the FC and control groups. In the three selected brain regions, partial least squares-discriminant analysis (PLS-DA) showed the variance in the data among five components (Fig. 2). In PLS-DA of PPC, 36.2% of the variance was explained by Component 1, 10% by Component 2, 10.1% by Component 3, 9.1% by Component 4, and 5.9% by Component 5 (Fig. 2a). In the AMG dataset, 27.2% of the variance was explained by Component 1, 15.8% by Component 2, 6.7% by Component 3, 6.2% by Component 4, and 3.8% by Component 5 (Fig. 2b). In HPC, 37.4% of the variance was explained by Component 1, 10.9% by Component 2, 5.2% by Component 3, 4.5% by Component 4, and 3.3% by Component 5 (Fig. 2c). The score plot for each brain region between Components 1 and 2 is shown in Fig. 2. PLS-DA indicated nearly perfect separations of FC and control groups in all brain regions. 3D score plots of the PLS-DA of PPC, AMG, and HPC are shown in Additional file 1: Fig. S1.

**Fig. 3:**
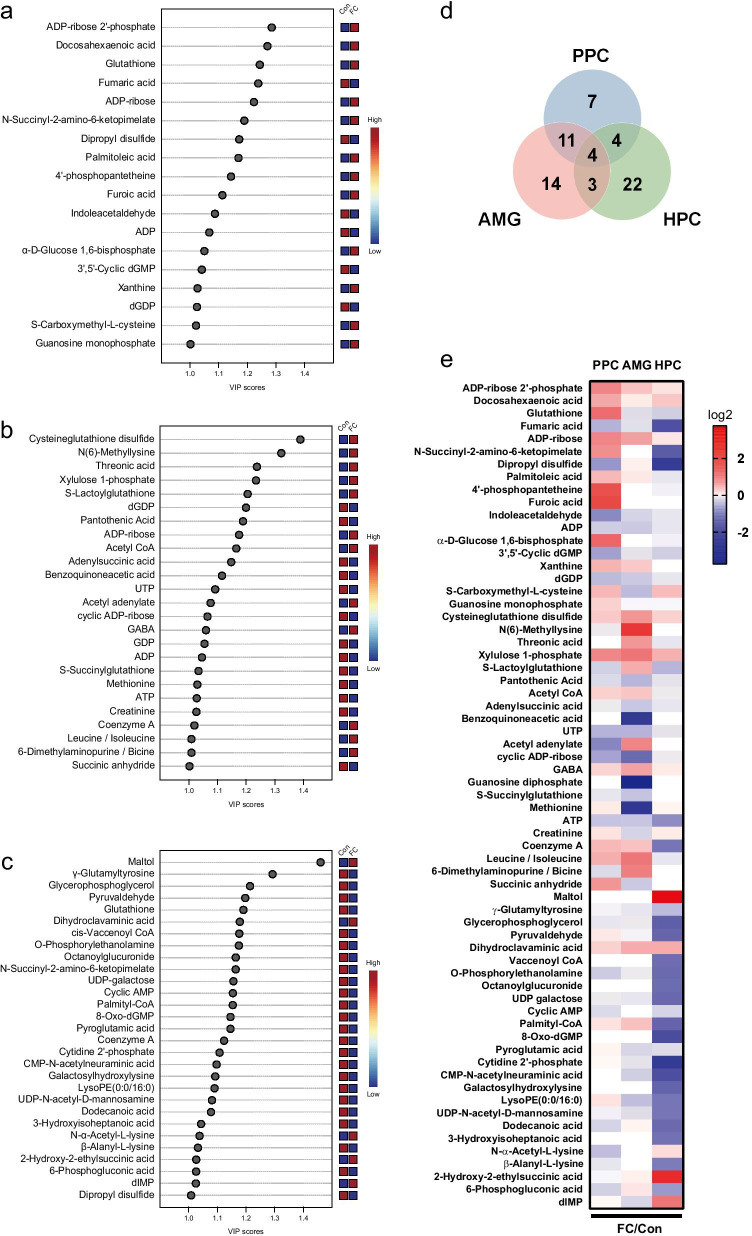
Metabolites selected on the basis of VIP score in fear retrieval. **a** Top metabolites (VIP score ≥ 1.0) in PPC. **b** Top metabolites (VIP score ≥ 1.0) in AMG. **c** Top metabolites (VIP score ≥ 1.0) in HPC. **d** Venn diagram showing the numbers of overlapping metabolites in each comparison. **e** Heat map of 65 metabolites selected on the basis of VIP score from the three brain regions. Rows and columns represent the brain regions and the 65 selected metabolites, respectively. Each cell is colored based on log 2 scale of the relative fold change in each brain region. *Con* control, *FC* fear conditioning, *VIP* variable importance in projection

To identify metabolites driving the separation between the FC and control groups, metabolites from each brain tissue were ranked by variable importance in projection (VIP) scores generated from the PLS-DA. Metabolites with high VIP scores (≥ 1.0) were considered contributing to observed separation; 18 fear memory–relevant metabolites with important variations were identified by ascribing VIP scores in PPC, 25 in AMG, and 29 in HPC. The VIP scores of metabolites in each brain region and the patterns of their changes are shown in Fig. 3a–c. On the basis of the VIP score ≥ 1.0, some overlapping metabolites tended to be increased and some tended to be decreased (*p*-value < 0.1, fold change ≥|1.1|) after fear retrieval in PPC, AMG, and HPC, as revealed by intersection analysis. Twenty-two metabolites were identified in more than one brain region. Fifteen metabolites differed between FC and control-group mice in both PPC and AMG, 8 in PPC and HPC, and 7 in AMG and HPC. Four metabolites overlapped among all three selected brain regions (Fig. 3d). A heat map in Fig. 3e illustrates the trends of changes in the relative levels of the 65 metabolites compared between FC and control groups in PPC, AMG, and HPC. Of note, 13 of the 15 metabolites overlapping between PPC and AMG had similar patterns of increases and decreases (Fig. 3e). The detailed data of metabolites such as fold changes and overlaps are provided in Additional file 2: Dataset S1.

### Metabolic pathway analysis in conditioned fear memory and identification of representative metabolites in PPC

**Fig. 4:**
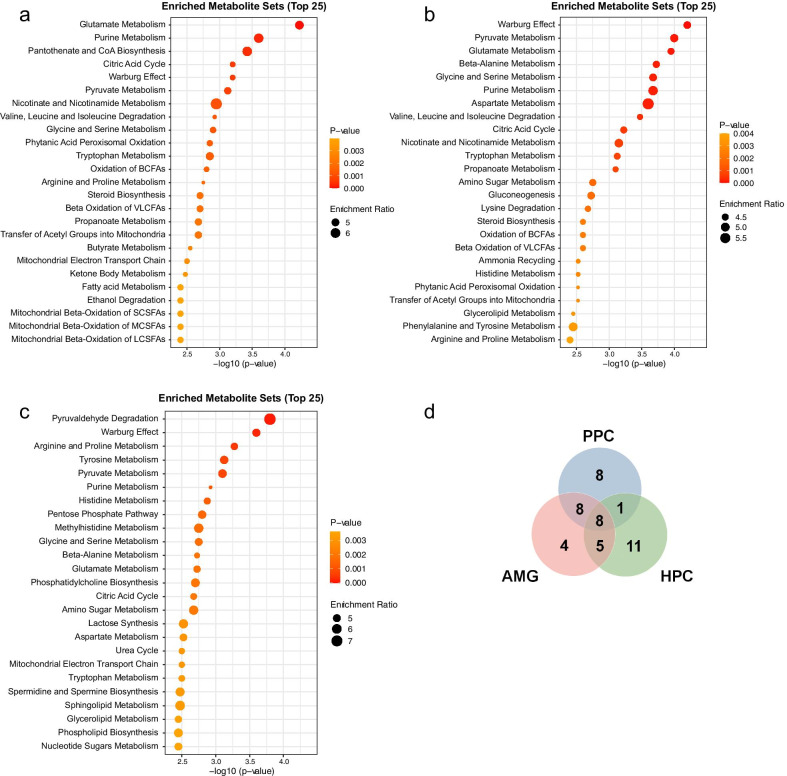
Metabolite set enrichment analysis of differential metabolites in three brain tissues. **a** Top pathways in PPC. **b** Top pathways in AMG. **c** Top pathways in HPC. **d** Venn diagram showing the numbers of overlapping metabolic pathways that differed between FC and Con in PPC, AMG, and HPC

To further determine the biological significance of the fear memory retrieval–relevant metabolites in each brain region, we performed a metabolite set enrichment analysis. Metabolites that were significantly changed were selected and the metabolic pathways that involve these metabolites were examined by enrichment analysis. Metabolites with *p*-values < 0.05 were selected for the enrichment analysis (26 in PPC, 35 in AMG, and 43 in HPC). Additionally, metabolites with *p*-values < 0.1 (10 in PPC, 14 in AMG, and 14 in HPC) were included in the list for the enrichment analysis. Thus, the datasets included 36 metabolites for PPC, 49 for AMG, and 57 for HPC. Enrichment analyses using these datasets identified the top 25 metabolic pathways contributing to the separation between FC and control groups (Fig. 4a–c). Details of these pathways are provided in Fig. 4 and Additional file 3: Dataset S2. On the basis of the overlap analysis, sixteen pathways overlapped between PPC and AMG, 9 between PPC and HPC, and 13 between AMG and HPC (Fig. 4d). Thus, more metabolic pathways, including metabolism of amino acids, purine metabolism, and oxidation of fatty acids, overlapped between PPC and AMG than in the other comparisons. The sets of metabolites that differed between FC and control groups in all three brain regions exhibited enrichment in 8 pathways: arginine and proline metabolism, citric acid cycle, glutamate metabolism, glycine and serine metabolism, purine metabolism, pyruvate metabolism, tryptophan metabolism, and Warburg effect (Fig. 4d and Additional file 3: Dataset S2). Among 22 overlapping metabolic pathways on comparing each brain tissue with all other brain tissues, 17 pathways with high ranks in PPC were identified (Additional file 3: Dataset S2). Therefore, we performed pathway analysis that integrated metabolite set enrichment analysis and pathway topology analysis using the datasets of PPC metabolites and KEGG database in MetaboAnalyst software to identify the impact value of each pathway and the importance value of each metabolite (Additional file 4: Fig. S2). The pathways with high significance (*p*-value < 0.01) and impact value, and the metabolites with *p*-values < 0.1 and importance value in these pathways are listed in Table 1.

**Table 1:** Pathway analysis of PPC metabolites altered in fear retrieval

Pathway name	*p*-value	FDR	Impact	Metabolites	Importance	FC	*p*-value
Pantothenate and CoA biosynthesis^a^	0.0003	0.007	0.4107	PPanSH	0.2357	3.18	0.0006
				CoA	0.1750	1.31	0.0677
Purine metabolism	0.0005	0.007	0.1366	GMP	0.0609	1.16	0.0186
				Xanthine	0.0297	1.36	0.0050
				ADP	0.0210	0.83	0.0114
				dGDP	0.0084	0.76	0.0398
				ATP	0.0070	0.79	0.0470
				Deoxyguanosine	0.0051	1.22	0.0156
				Adenine	0.0046	1.33	0.0316
				ADPR	0.0000	1.79	< 0.0001
Tryptophan metabolism	0.0012	0.012	0.0139	Indoleacetaldehyde	0.0139	0.57	0.0036
				Acetyl-CoA	0.0000	1.16	0.0184
Pyruvate metabolism	0.0017	0.013	0.1540	Acetyl-CoA	0.1540	1.16	0.0184
				Acetyl adenylate	0.0000	0.55	0.0145
				Fumarate	0.0000	0.72	0.0595
Glutathione metabolism^a^	0.0066	0.029	0.2560	Glutathione	0.2560	2.22	0.0444
				Acetyl-CoA	0.0000	1.16	0.0184
Citrate cycle (TCA cycle)	0.0081	0.029	0.0992	Acetyl-CoA	0.0367	1.16	0.0184
				Succinate	0.0327	1.14	0.0977
				Fumarate	0.0298	0.72	0.0595

*FC* fold change, *FDR* false discovery rate, *ADPR* ADP-ribose, *PPanSH* 4′-Phosphopantetheine

^a^Pathway with high impact score (> 0.2)

In PPC, pantothenate and CoA biosynthesis had the highest significance and impact value. 4′-Phosphopantetheine had the highest significance and importance value in this pathway. Purine metabolism had a high significance level in pathway analysis. Various metabolites were significantly changed in purine metabolism, although each metabolite had a relatively low importance value. Glutathione metabolism had comparatively high significance and impact value. Glutathione had a high importance value in this pathway (Table 1). ADP-ribose (ADPR), a major metabolite in NAD^+^-dependent signaling, had the highest significance among all metabolites in PPC. Other metabolites with significance (*p*-value < 0.05), such as ADP-ribose 2′-phosphate (ADPRP; *p*-value < 0.0001), cyclic ADP-ribose (cADPR; *p*-value = 0.0275), and NADH (*p*-value = 0.0199) are also involved in NAD^+^-dependent signaling. Therefore, NAD^+^-dependent signaling seems to be one of the pathways perturbed by fear retrieval in PPC.

**Fig. 5:**
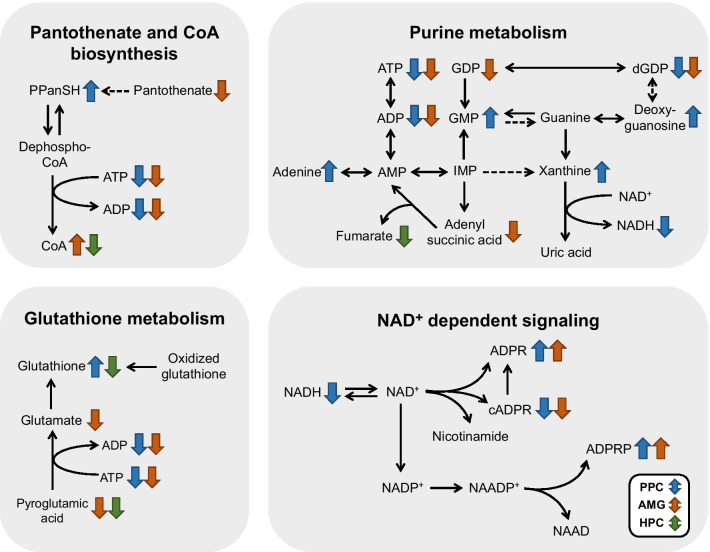
The perturbed major metabolic pathways of PPC and significantly different metabolites in the fear retrieval condition. Blue arrows, significantly changed metabolites of PPC; red arrows, significantly changed metabolites of AMG; green arrows, significantly changed metabolites of HPC. *PPanSH* 4′-Phosphopantetheine, *ADPR* ADP-ribose, *ADPRP* ADP-ribose 2′-phosphate, *cADPR* cyclic ADP-ribose, *NAADP*^*+*^ nicotinic acid adenine dinucleotide phosphate, *NAAD* nicotinic acid adenine dinucleotide

**Fig. 6:**
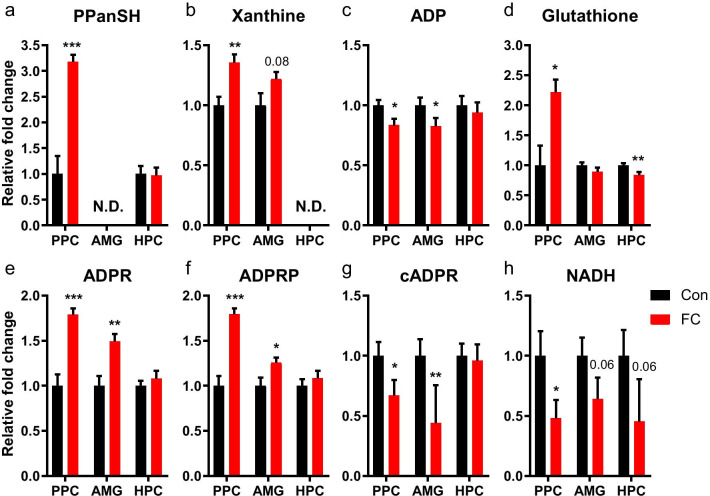
Changes of representative metabolites in the most perturbed metabolic pathways. Each metabolite was presented with *p*-value < 0.05, fold change ≥|1. 2|, and high importance on each pathway in PPC. The fold change and statistical significance of each metabolite for FC vs. Con in PPC, AMG, and HPC were presented (Con-PPC, n = 16 mice; FC-PPC, n = 17 mice; Con-AMG, n = 16 mice; FC-AMG, n = 17 mice; Con-HPC, n = 16 mice; FC-HPC, n = 13 mice). Two-tailed unpaired *t*-test, data are mean ± SEM; *p < 0.05, **p < 0.01, ***p < 0.001. *N.D.* not determined. *PPanSH* 4′-Phosphopantetheine, *ADPR* ADP-ribose, *ADPRP* ADP-ribose 2′-phosphate, *cADPR* cyclic ADP-ribose

Analysis of significantly altered metabolites and pathways prompted us to depict representative pathways (Fig. 5). Pantothenate and CoA biosynthesis, purine metabolism, glutathione metabolism, and NAD^+^-dependent signaling were the pathways most perturbed in PPC by fear memory retrieval. Major metabolites of these pathways were significantly regulated in PPC with *p*-values < 0.05 and fold change ≥|1. 2|. Fear memory retrieval increased the level of 4′-phosphopantetheine, xanthine, and glutathione and decreased the level of ADP in PPC (Fig. 6a-d). In AMG, the level of xanthine tended to increase and ADP level decreased significantly, similar to PPC (Fig. 6b and c). In contrast, glutathione level was significantly reduced in HPC (Fig. 6d). In PPC, ADPR and ADPRP were increased, whereas cADPR and NADH were decreased by fear memory retrieval (Fig. 6e–h). Fear retrieval also increased the level of ADPR and ADPRP but decreased the level of cADPR in AMG (Fig. 6e–g). Notably, the trends of changes in NADH in both AMG and HPC were similar to that in PPC (Fig. 6h). Those metabolites were significantly regulated and could be used as biomarkers specific to conditioned fear memory in PPC.

## Discussion

Our study revealed that fear memory retrieval significantly changed PPC, AMG, and HPC metabolites and perturbed related metabolic pathways. Conditioned fear memory is a risk factor for mental disorders such as anxiety, depression, and PTSD [36, 37]. Numerous studies have investigated metabolism underlying various stress-related psychiatric disorders by using models including conditioned fear memory in AMG and HPC. For example, in a rat model of chronic unpredictable mild stress (a commonly used model of depression), most differential metabolites in HPC were related to amino acid metabolism including that of glutamine and to lipid metabolism including that of cholesterol, although some metabolites in glucose metabolism and the tricarboxylic acid cycle were also significantly changed [38]. In the HPC of mice exposed to a different chronic unpredictable mild stress condition, the levels of urea, phosphoric acid, glutamine, and cholesterol were increased and those of N-carboxy-glycine, hexadecanoic acid, and octadecanoic acid were decreased [39]. Purine metabolism in HPC was perturbed in both chronic social defeat stress and fear conditioning mouse models [35, 40]. In the HPC of susceptible mice that received chronic social defeat stress, the hyperfunction of the fatty acid beta-oxidation cycle was also shown [35]. Overall, various metabolic pathways including amino acid, lipid, carbohydrate, and purine metabolism are affected in HPC in diverse stress models. Other studies suggest that AMG metabolites are also altered in several stress conditions associated with developing mental disorders. Perturbation in amino acid, fatty acid, and carbohydrate metabolism was observed in the AMG in the chronic unpredictable mild stress rat model, similar to the findings in HPC [41]. The level of the amino acid serine was significantly decreased in the AMG of dominant hamsters following a social defeat encounter [34]. Our study showed that various metabolites in amino acid, carbohydrate, and purine metabolism were changed in the AMG and HPC in the conditioned fear model. Especially, lipid metabolisms including phosphatidylcholine biosynthesis and sphingolipid metabolism were significantly regulated in the HPC.

Some studies have investigated the metabolites and pathways that are differentially regulated in the PFC and nucleus accumbens as well as AMG and HPC in social defeat stress and chronic restraint stress models of mental disorders [34, 42]. Although the PPC is implicated in fear learning and memory [9, 29], the metabolites and metabolic pathways that are affected in PPC remain unknown. In our study, UPLC-MS/MS based metabolomics analysis data showed that purine metabolism, pantothenate and CoA biosynthesis, glutathione metabolism, and NAD^+^-dependent signaling were the most perturbed pathways in the retrieval of conditioned fear memory.

Our study suggests that pantothenate and CoA biosynthesis, which had the highest significance and impact value in pathway analysis, was the most perturbed pathway in PPC by conditioned fear memory. In pantothenate and CoA biosynthesis, pantothenate is phosphorylated by pantothenate kinase and converted to 4'-phosphopantetheine, an intermediate of this pathway [43]. Pantothenate kinase KO mice displayed increases in gene expressions of dopamine D1 receptor (D1R) and dopamine D2 receptor (D2R) in globus pallidus-containing region including striatum [44]. Interestingly, local infusion of D1R antagonist SCH23390 into AMG, HPC or PFC prior to the fear conditioning impairs fear responses such as freezing [45, 46]. Dentate gyrus-specific D1R KO mice showed significant fear memory deficits [47]. To test the change in gene expression of *D1r* in PPC, we measured and compared the mRNA level of *D1r* in PPC of the control and FC groups. The mRNA level of *D1r* was significantly increased in the FC group (Additional file 5: Fig. S3) whereas those of other proteins such as tyrosine hydrolase and D2R related to dopamine metabolism were barely detected in PPC (data not shown). It is possible that the perturbed pantothenate and CoA biosynthesis pathway upregulates *D1r* expression in PPC and the increase in *D1r* might be involved in fear responses as in the AMG, HPC or PFC.

In our data, purine metabolism included most metabolites that were changed by fear retrieval in PPC. Xanthine might be important among the significantly altered metabolites in this pathway. Xanthine is formed by the oxidation of hypoxanthine, and the intra-striatal administration of hypoxanthine impairs fear memory learning, consolidation, and retrieval [48]. One paper showed that electronic foot shock increased serum Xanthine [49]. Xanthine was significantly increased in PPC but was not detected in HPC. In AMG, the trend of changes in the level of xanthine was similar to that in PPC. Additionally, ADP level was significantly decreased in PPC and AMG. Since ADP is related to various metabolic pathways, further studies of its functions and related pathways in the regulation of fear memory in PPC are needed.

In glutathione metabolism, we found that the level of glutathione was increased in PPC and decreased in HPC. Glutathione is an anti-oxidant, and an increase in glutathione levels plays a role in protection from oxidative stress [50]. The level of oxidative stress is reportedly elevated in a PTSD model [51]. One study suggested that the level of cystine, which is converted to cysteine, a precursor to glutathione, increases under social defeat stress [34]. The sensitivity to oxidative stress varies in different brain regions. It was suggested that HPC may be a sensitive target of toxic oxidative bursts compared to other brain regions in Carioca high-conditioned freezing animals which present high defensive freezing responses to contextual fear [52]. Therefore, glutathione may be rapidly oxidized by oxidizing agents. As such, our study shows that glutathione levels seem to be decreased by conditioned fear-induced oxidative stress in HPC. In contrast, the increased glutathione levels of PPC might be due to a protective mechanism against oxidative stress, suggesting that PPC may be less sensitive to oxidative stress compared to HPC.

Several studies have demonstrated that various enzymes including CD38, CD157, and SARM1 produce both cADPR and ADPR using NAD^+^ as substrate [53–56]. CD38 also hydrolyzes cADPR to ADPR and nicotinic acid adenine dinucleotide phosphate to ADPRP [56, 57]. These enzymes are involved in fear conditioning and fear memory retrieval. CD38 is important in fear learning processes including fear acquisition, extinction, and retrieval [58, 59]. CD157 is expressed in the AMG and regulates anxiety-related and depression-like behaviors including fear response [60]. SARM1 is also expressed in the AMG and has roles in both HPC- and AMG-dependent fear conditioning [61]. Thus, our results suggest that changes in metabolites such as ADPR, cADPR, ADPRP, and NADH are associated with NAD^+^-related metabolism and the perturbed metabolic pathway in PPC and AMG might be involved in the conditioned fear memory.

Future comprehensive metabolomics analyses using various models such as fear extinction and fear renewal may pave the way toward better understanding of the significance of metabolites and metabolic pathways in fear memory regulation including destabilization, reconsolidation, and extinction after fear memory retrieval, thereby providing ideas for therapeutic strategies for the treatment of fear-related mental disorders.

## Conclusion

We analyzed and compared changes of metabolites in the HPC, AMG, and PPC in conditioned fear memory. One of the purposes of this study was to identify the metabolites that could be exploited for the development of potential biomarkers for fear-related mental illnesses. While our analysis of fear memory–induced changes in metabolites within selected brain regions indicated that many small molecules are changed significantly between FC and control mice, our study identified 4′-phosphopantetheine, xanthine, glutathione, ADPR, cADPR, and ADPRP as putative biomarkers and suggests that the corresponding metabolic pathways are related to fear memory in PPC.

## Methods

### Animals

Male C57BL/6 mice (8 weeks old; purchased from The Koatech) were maintained in a controlled-light environment (12 h light and 12 h dark cycle; lights on from 7 am to 7 pm) with ad libitum access to standard laboratory chow (Teklad 2018) and water. Mice were handled daily for 1 week prior to behavioral procedures for fear conditioning and retrieval tests. All behavioral procedures were performed in accordance with the guidelines on care and use of laboratory animals as approved by the DGIST Institutional Animal Care and Use Committee to minimize animal suffering.

### Fear conditioning procedure

The fear conditioning procedure was performed in accordance with a previous protocol [9]. For the retrieval test, the context and day conditions of the procedure were modified because fear extinction training was not required.

The mouse test cage (17.78 × 17.78 × 30.48 cm; Coulbourn Instruments) used for fear conditioning consisted of gray walls and a stainless-steel shock grid floor. The grid floor was composed of bars that allowed the delivery of electric foot shocks. Before fear conditioning and the retrieval test, the wall and floor were cleaned with 75% ethanol.

On the day of fear conditioning, mice in the FC group were acclimated in the test cage for 300 s, and a tone (CS; 2.8 kHz, 85 dB, 30 s) was then co-terminated with an aversive foot shock (US; 0.2 mA, 0.5 s), which was presented 6 times at 300, 390, 500, 630, 740, and 870 s [62]. The variable time interval between trials which are tones co-terminated with a foot shock was used to minimize the possibility of the animals expecting the next trial. The retrieval test was performed on the next day. After exploration for 300 s, the same tone, without an electric foot shock, was played twice, at 300 and 390 s. The mice in the control group were exposed to the same context and tones at the same times as in the FC group without receiving foot shocks. Freezing is defined as the suppression of movement with the exception of respiration. FreezeFrame software (Coulbourn Instruments) was used to score freezing behavior, defined as mouse immobility lasting more than 1 s in accordance with a reference [9].

### Tissue collection

After behavioral procedures, the mice were anesthetized with isoflurane, and brains were collected. Brains were sliced coronally at 1 mm thickness by using a brain matrix. The PPC and AMG punches (2.0 mm diameter for PPC and 1.5 mm diameter for AMG) were collected bilaterally from a slice section (1.0 mm; from − 1.0 to − 2.0 mm relative to bregma). The HPC tissues were manually dissected and cut bilaterally in two slice section (1.0 mm each; from − 1.0 to − 3.0 mm relative to bregma). The tissues were flash frozen in liquid nitrogen and stored at − 80 °C until metabolite extraction. In some brain regions, tissue was not assayed because of inaccurate dissections.

### Sample preparation

PPC and AMG tissues were extracted with 500 µl of Chilled extraction solvent (acetonitrile:methanol:water, 2:2:1, v/v) containing 5 µM ATP-^13^C_10_ (Sigma-Aldrich), which was used as an internal standard. HPC tissue was extracted with 800 µl of the same extraction solvent with the internal standard. The mixtures were sonicated for about 7 min (10 cycles), vortexed for 10 min, and then incubated at − 20 °C for 1 h. The samples were centrifuged at 18,000×*g* for 10 min at 4 °C. The supernatants were collected and stored at − 20 °C. The pellets were re-extracted using 200 µl aliquots of the extraction solvent and the procedure was repeated. The supernatants were combined and dried under nitrogen flow in a nitrogen evaporator. The dry extracts were reconstituted with 100 µl of 60% acetonitrile (ACN) and filtered through a 0.22 µm PTFE filter before analysis.

### UPLC-MS analysis for data acquisition

All samples were analyzed using an Agilent 1290 UPLC system coupled to an Agilent 6530 Q-TOF MS (Agilent Technologies) in positive or negative ESI mode. The instrument parameters were as follows: gas temperature 325 °C, drying gas 11 l/min, nebulizer 30 psig, fragment voltage 170 V, skimmer 60 V, and capillary voltage ± 3,500 V (nozzle voltage 500 V; only negative mode). Internal mass calibration was performed using reference masses during the runs.

The sample (10 µl) was injected into a Merck ZIC-cHILIC column (100 × 2.1 mm, 3 µm) heated to 40 °C in a column oven. The mobile phase A consisted of 9/1 (v/v) water/ACN containing 10 mM ammonium acetate (pH 6.9) and the mobile phase B consisted of 1/9 (v/v) water/ACN containing 10 mM ammonium acetate and 0.1% acetic acid (pH 7.3). Flow rate was 0.3 ml/min and the gradient program was as follows: 0–20 min, 87.5–37.5% B; 20–21 min, 37.5%; column wash: 21–21.5 min, 37.5–0% B; 21.5–23.5 min, 0% B; 23.5–24 min 0–87.5% B; the initial condition (87.5% B) was maintained for 7 min for equilibration. Untargeted data acquisition on the UPLC-MS was performed using Agilent MassHunter Workstation Data Acquisition software (Agilent Technologies).

### Data filtering and visualization

The UPLC-MS data were deconvoluted into individual chemical peaks and used to find a “feature” that describes a chemical entity in a chromatogram via the algorithms “Molecular Feature Extractor” and “Find by Formula” in Agilent MassHunter Qualitative Analysis B.07.00 software (Agilent Technologies). The results were used for subsequent statistical analysis and data visualization in Mass Profiler Professional (MPP) software (Agilent Technologies). To create feature lists, the data were filtered on the basis of the number of entities detected in at least one condition (FC or control) in each brain region. Features appearing in more than half of the samples in at least one group were used for subsequent analyses.

PLS-DA was performed to display the variance in samples within the groups. To generate the PLS-DA data, the MetaboAnalyst web tool (www.metaboanalyst.ca) was used. All data were normalized by using an auto-scaling method that attempts to scale and center the data by dividing each value by standard deviation. By extracting the PLS-DA score values, the visualization aid was generated with a 95% confidence interval. VIP score plots were also generated from the PLS-DA function of MetaboAnalyst. After PLS-DA, the fold change data of metabolites with high VIP scores were log 2 transformed and then displayed as a heat map in GraphPad Prism 8 software. For visual comparison, the concentration changes in the heat map were shown using a color scale ranging from + 3 (red) to − 3 (blue).

### UPLC-MS/MS analysis for identification of features

The metabolic features that differed between FC and control were identified by accurate mass and isotopic abundance pattern matching to the METLIN database in MPP. To confirm the identity of these metabolites, UPLC-MS/MS analysis was subsequently performed. The lists of compounds created in MPP, which included *m*/*z* and retention time values, were used as targets in MS/MS analysis. Several brain samples were re-analyzed by targeted MS/MS analysis on the Q-TOF MS. The sample sizes of PPC, AMG, and HPC for UPLC-MS/MS are 25, 26, and 29, respectively. MS/MS spectra were generated at a given collision energy and the sample spectra were matched to a MS/MS spectral library of standards in METLIN database by using Agilent MassHunter Molecular Structure Correlator B.05.00 software (Agilent Technologies).

### Pathway analysis

Metabolite set enrichment analyses in the three brain regions were performed using MetaboAnalyst. The analyses were based on SMPDB libraries containing 99 groups of metabolite sets that are linked to KEGG database and HMDB. To determine the significance of changes in metabolites and related pathways revealed by enrichment analysis, pathway analysis of PPC was performed using KEGG database in MetaboAnalyst. The pathway analysis module combines results from enrichment analysis and pathway topology analysis.

### Real-time quantitative PCR

Total RNA from brain tissue was isolated with TRIzol reagent (Invitrogen) according to the manufacturer’s instructions. The concentration of RNA was determined using a NanoDrop spectrophotometer (Thermo Scientific). cDNA was synthesized from 1.6 µg RNA using the GoScript™ Reverse Transcription System (Promega) as described previously [63]. In a CFX 96 Real-Time system (Bio-Rad), TB Green (TaKaRa Biotechnology) was used for quantitative PCR estimation of the expression of *D1r* and *Gapdh* genes. The following primer pairs were used: *D1r*, forward primer, 5′-CAGCCTTCATCCTGATTAGCGTAG-3′, reverse primer, 5′-CTTATGAGGGAGGATGAAATGGCG-3′. *Gapdh*, forward primer, 5′-ATCACTGCCACCCAGAAGAC-3′, reverse primer, 5′-ACACATTGGGGGTAGGAACA-3′. The expression level was normalized to that of *Gapdh* as an endogenous control.

### Statistical analysis

Data were analyzed using univariate and multivariate analyses, including unpaired *t*-test for independent pairs of groups. The effects of fear retrieval on classification of the samples of each brain tissue were compared. A cut-off value of asymptotic *p* < 0.05 and threshold > 1.1 were considered significant in unpaired *t*-test using MPP software. The pathway analyses were performed and the *p*-value and impact value were assessed using MetaboAnalyst software. All data are presented as mean ± standard error of the mean (SEM). For comparisons of data between two groups, the two-tailed unpaired *t*-test was used. For comparing freezing percentage data of more than three groups, one-way ANOVA followed by Tukey’s multiple comparisons or two-way repeated-measures ANOVA was performed using GraphPad Prism 8 Software. A *p*-value of < 0.05 was considered statistically significant.

## Supplementary Information

**Additional file 1: Fig. S1.** The 3D score plots of PLS-DA in three brain tissues. **a** PPC. **b** AMG. **c** HPC (Con-PPC, n = 16 mice; FC-PPC, n = 17 mice; Con-AMG, n = 16 mice; FC-AMG, n = 17 mice; Con-HPC, n = 16 mice; FC-HPC, n = 13 mice).

**Additional file 2: Dataset S1.** Details of statistical analysis and fold changes of overlapping metabolites in Fig. 3d and e. Significant increases in fold change value are highlighted in red and significant decreases are highlighted in blue. FC, fold change; N.D., not determined.

**Additional file 3: Dataset S2.** Details of enrichment analysis in three brain regions in Fig. 4. Gray boxes, metabolic pathways overlapping between all three brain regions; pink boxes, metabolic pathways overlapping between PPC and AMG; orange boxes, metabolic pathways overlapping between AMG and HPC; green boxes, metabolic pathways overlapping between PPC and HPC; white boxes, metabolic pathways identified in a single brain region. FDR, false discovery rate.

**Additional file 4: Fig. S2.** Pathway analysis of metabolites changed by fear retrieval in PPC.

**Additional file 5: Fig. S3.** The relative mRNA level of the dopamine D1 receptor in PPC (Con, n = 6 mice; FC, n = 6 mice). Two-tailed unpaired *t*-test, data are mean ± SEM; *p < 0.05.

## Data Availability

All data generated or analyzed during this study are included in this published article.

## Abbreviations

ACN: Acetonitrile
ADPR: ADP-ribose
ADPRP: ADP-ribose 2′-phosphate
AMG: Amygdala
cADPR: Cyclic ADP-ribose
CS: Conditioned stimulus
D1R: Dopamine D1 receptor
D2R: Dopamine D2 receptor
FC: Fear conditioning
FDR: False discovery rate
HPC: Hippocampus
MPP: Mass profiler professional
PFC: Prefrontal cortex
PLS-DA: Partial least squares discriminant analysis
PPC: Posterior parietal cortex
PTSD: Post-traumatic stress disorder
UPLC-MS: Ultra-performance liquid chromatography–mass spectrometry
UPLC-MS/MS: Ultra-performance liquid chromatography–tandem mass spectrometry
US: Unconditioned stimulus
VIP: Variable importance in projection
