# Supplementary figures and images for: Comparative metabolic profiling of posterior parietal cortex, amygdala, and hippocampus in conditioned fear memory
Source: Mol Brain. 2021 Oct 6;14:153. doi: 10.1186/s13041-021-00863-x (PMC8493686; doi:10.1186/s13041-021-00863-x)

a

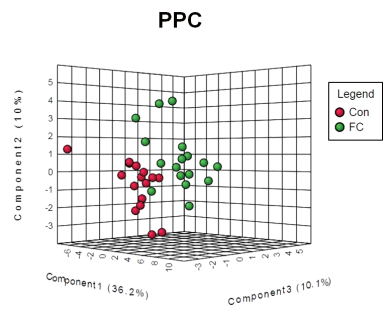

b

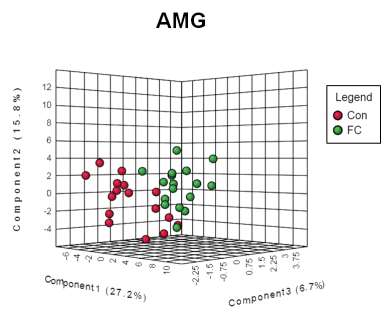

c

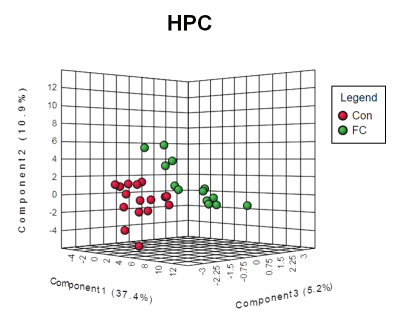

Supplement: Supplementary file 1 — Additional file 1: Fig. S1. The 3D score plots of PLS-DA in three brain tissues. a PPC. b AMG. c HPC (Con-PPC, n = 16 mice; FC-PPC, n = 17 mice; Con-AMG, n = 16 mice; FC-AMG, n = 17 mice; Con-HPC, n = 16 mice; FC-HPC, n = 13 mice). [file 13041_2021_863_MOESM1_ESM.pdf]

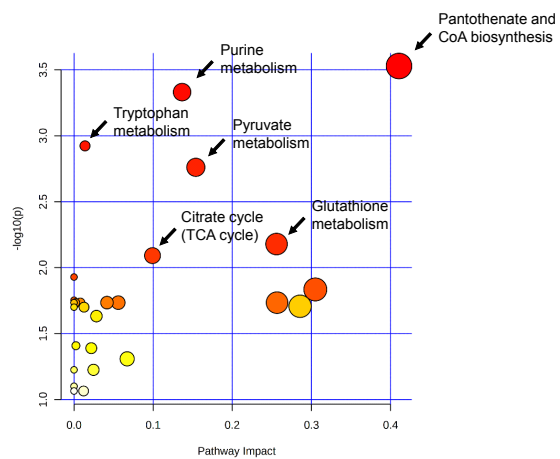

Supplement: Supplementary file 4 — Additional file 4: Fig. S2. Pathway analysis of metabolites changed by fear retrieval in PPC. [file 13041_2021_863_MOESM4_ESM.pdf]

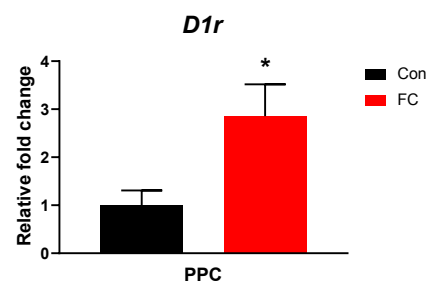

Supplement: Supplementary file 5 — Additional file 5: Fig. S3. The relative mRNA level of the dopamine D1 receptor in PPC (Con, n = 6 mice; FC, n = 6 mice). Two-tailed unpaired t-test, data are mean ± SEM; *p < 0.05. [file 13041_2021_863_MOESM5_ESM.pdf]
